# Updates on Hearing From the Global Burden of Disease Study

**DOI:** 10.1093/geroni/igaa057.2934

**Published:** 2020-12-16

**Authors:** Lydia Haile, Aislyn Orji, Paul Briant, Jaimie Adelson, Adrian Davis, Theo Vos

**Affiliations:** 1 University of Washington, Seattle, Washington, United States; 2 Institute for Health Metrics and Evaluation, Seattle, Washington, United States; 3 Imperial College London, London, England, United Kingdom

## Abstract

Hearing loss is estimated annually as part of the Global Burden of Disease study. In 2019, input data came from 308 surveys in 77 countries. Hearing loss was measured by taking the pure-tone average of audiometric thresholds ranging from .5– 4 kHz. We ran severity-specific prevalence models, adjusting the severity for hearing aid use down one level, and separately estimating hearing loss due to meningitis, otitis media, congenital abnormalities, and age-related and other factors. In 2019, 1.5 billion (95% UI=1.5–1.6) people experienced hearing loss, primarily due to age and other factors. Of those with hearing loss, 74% (UI=71–77%) had mild hearing loss and 7% (UI=6–9%) used hearing aids. Globally, age-related hearing loss was the third-ranked cause of Years Lived with Disability after low back pain and migraine. Due to ageing, the hearing loss burden has increased over time, indicating a greater need for hearing healthcare services world-wide.

